# Physicians’ Experiences With Mistreatment and Discrimination by Patients, Families, and Visitors and Association With Burnout

**DOI:** 10.1001/jamanetworkopen.2022.13080

**Published:** 2022-05-19

**Authors:** Liselotte N. Dyrbye, Colin P. West, Christine A. Sinsky, Mickey Trockel, Michael Tutty, Daniel Satele, Lindsey Carlasare, Tait Shanafelt

**Affiliations:** 1 Mayo Clinic, Rochester, Minnesota; 2 now with University of Colorado School of Medicine, Denver; 3 American Medical Association, Chicago, Illinois; 4 Psychiatry and Behavioral Sciences, Stanford School of Medicine, Stanford, California; 5 Department of Health Sciences Research, Mayo Clinic, Rochester, Minnesota; 6 Stanford School of Medicine, Stanford, California

## Abstract

**Importance:**

Burnout is common among physicians and is associated with suboptimal patient outcomes. Little is known about how experiences with patients, families, and visitors differ by physician characteristics or contribute to the risk of burnout.

**Objective:**

To examine the occurrence of mistreatment and discrimination by patients, families, and visitors by physician characteristics and the association between such interactions and experiencing burnout.

**Design, Setting, and Participants:**

This cross-sectional survey was conducted from November 20, 2020, to March 23, 2021, among US physicians.

**Exposures:**

Mistreatment and discrimination were measured using items adapted from the Association of American Medical College’s Graduation Questionnaire with an additional item querying respondents about refusal of care because of the physicians’ personal attributes; higher score indicated greater exposure to mistreatment and discrimination.

**Main Outcomes and Measures:**

Burnout as measured by the Maslach Burnout Inventory.

**Results:**

Of 6512 responding physicians, 2450 (39.4%) were female, and 369 (7.2%) were Hispanic; 681 (13.3%) were non-Hispanic Asian, Native Hawaiian, or Pacific Islander; and 3633 (70.5%) were non-Hispanic White individuals. Being subjected to racially or ethnically offensive remarks (1849 [29.4%]), offensive sexist remarks (1810 [28.7%]), or unwanted sexual advances (1291 [20.5%]) by patients, families, or visitors at least once in the previous year were common experiences. Approximately 1 in 5 physicians (1359 [21.6%]) had experienced a patient or their family refusing to allow them to provide care because of the physician’s personal attributes at least once in the previous year. On multivariable analyses, female physicians (OR, 2.33; 95% CI, 2.02-2.69) and ethnic and racial minority physicians (eg, Black or African American: OR, 1.59; 95% CI, 1.13-2.23) were more likely to report mistreatment or discrimination in the previous year. Experience of mistreatment or discrimination was independently associated with higher odds of burnout (vs score of 0 [no mistreatment], score of 1: OR, 1.27; 95% CI, 1.04-1.55; score of 2: OR, 1.70; 95% CI, 1.38-2.08; score of 3: OR, 2.20; 95% CI, 1.89-2.57). There was no difference in the odds of burnout by gender after controlling for experiencing mistreatment and discrimination score and other demographic factors, specialty, practice setting, work hours, and frequency of overnight call.

**Conclusions and Relevance:**

In this study, mistreatment and discrimination by patients, families, and visitors were common, especially for female and racial and ethnic minority physicians, and associated with burnout. Efforts to mitigate physician burnout should include attention to patient and visitor conduct.

## Key Points

**Question:**

How frequently do physicians experience mistreatment and discrimination by patients, their families, and visitors; how does this vary by physician characteristics; and what is the association between having such interactions and experiencing burnout?

**Findings:**

In this cross-sectional study of 6512 US physicians, mistreatment and discriminatory behaviors by patients, families, and visitors within the previous year were common, especially for female and racial and ethnic minority physicians, and associated with higher burnout rates.

**Meaning:**

The findings suggest that efforts to mitigate risk of physician burnout and improve the work experience of female and racial and ethnic minority physicians should include strategies that promote patient, family, and visitor civility.

## Teaser

This cross-sectional study examines the occurrence of mistreatment of and discrimination toward physicians by patients, families, and visitors by physician characteristics and the association between such interactions and experiencing burnout.

## Introduction

Burnout is an occupational phenomenon characterized by emotional exhaustion, cynicism related to one’s work, and reduced sense of professional efficacy that is prevalent among physicians. Studies have linked burnout to detrimental professional and personal consequences.^1,2^

Burnout occurs due to high work stress^3^ and may be caused, in part, by negative interactions with patients, families, and visitors. Prior studies have shown that physicians experience verbal and physical mistreatment and discrimination at work due to their visible personal attributes.^4,5,6,7,8,9,10,11,12^ These studies have primarily focused on the perpetrator of mistreatment being another health care worker, have been conducted in a single specialty or outside the United States, or did not differentiate the potential impact of the experience by the type of perpetrator (institutional employee vs patient, family member, or visitor).^6,9,13,14,15,16,17,18,19^

Little is known about mistreatment and discrimination experiences of physicians by personal characteristics (eg, gender and race) and professional characteristics (eg, specialty, practice setting) or the potential professional impact of mistreatment and discrimination by patients, families, and visitors.^5^ Therefore, we studied the prevalence of physicians experiencing mistreatment and discrimination originating from patients, their families, and visitors and explored the association between such experiences and occupational burnout.

## Methods

### Participants

As previously described,^20^ we conducted a multimodality survey of physicians in the AMA Physician Masterfile: a sample of 4000 physicians who were mailed a paper survey and a $20 check as well as a sample of 90 000 physicians who received an electronic survey without incentive (to increase number of participants for analysis).^20^ Additional details on sampling can be found in the eAppendix in the Supplement. Briefly, both samples included physicians from all specialties with oversampling of physicians not specializing in general internal medicine, general pediatrics, family medicine, and obstetrics and gynecology to increase the sample size of physicians from smaller specialties. Of the 4000 who were mailed a survey, 329 were undeliverable, leaving a final sample of 3671. Invitations to participate were sent in November 2020. For this analysis, we included responders to the mailed or electronic survey who completed the mistreatment and discrimination survey items described in the next section. A secondary survey of nonresponders indicated participants were representative with respect to demographic characteristics, single-item emotional exhaustion or depersonalization scores, overall burnout prevalence, and satisfaction with work-life integration.^20^

Participation was voluntary, and responses were anonymous. Informed consent was implied upon completing the survey. The Mayo Clinic and Stanford institutional review boards approved the study, and we followed the Strengthening the Reporting of Observational Studies in Epidemiology (STROBE) reporting guidelines.

### Study Measures

Both the paper and the electronic versions of the primary survey as well as the secondary survey of nonresponders contained items about age, gender, years in practice, burnout, and satisfaction with work-life integration. The primary survey contained additional items pertaining to personal factors (ie, race, ethnicity [Hispanic or Latino/a origin], relationship status) and professional factors (work hours per week, nights on call per week, specialty, practice setting, and experiences of mistreatment and discrimination at work) factors. The question on racial background provided the following choices: American Indian or Alaska Native, Asian, Black or African American, Pacific Islander or Native Hawaiian, White, and other; individuals could mark all that apply. For analysis, responses to the race and ethnicity questions were combined as follows: Hispanic or Latino/a, non-Hispanic Asian, Pacific Islander, or Native Hawaiian, non-Hispanic Black, non-Hispanic Indigenous or other, non-Hispanic White, and non-Hispanic 2 or more races.

#### Mistreatment and Discrimination

Using an iterative process involving all authors, we selected and modified items pertaining to mistreatment and discrimination experiences from the Association of American Medical Colleges (AAMC) Graduation Questionnaire (GQ) survey. Of the 17 AAMC GQ survey items on behaviors personally experienced during medical school, we selected 5 items most pertinent to practicing physicians and made several modifications. First, in contrast to the AAMC GQ items, which are intended to measure the frequency of certain behaviors exhibited by faculty, nurses, trainees, and other institution employees or staff, we specifically prompted respondents to only consider behaviors exhibited by patients, families, and visitors. Second, we modified the question stems by adding “at work” at the end of each statement. Third, we asked respondents to report behaviors personally experienced over the previous year (rather than “during medical school”). Finally, we changed the response categories from never, once, occasionally, and frequently to never, once, several times a year, weekly, and several times a week to help ensure respondents interpreted the response options similarly. An additional item explored the frequency with which respondents had experienced a patient or their family refusing to allow them to provide care for the patient because of visible personal attributes.^15^ Other studies inquiring about patient interactions have considered refusal of care as a form of discrimination.^5,8^ The aggregate mistreatment items are available in eTable 1 in the Supplement.

#### Burnout

Burnout was measured using the 9-item emotional exhaustion and 5-item depersonalization subscales of the Maslach Burnout Inventory (MBI) under license from Mind Garden, Inc. Physicians with high emotional exhaustion (≥27) and/or high depersonalization (≥10) scores were considered to have symptoms of burnout.^21^

### Statistical Analysis

In addition to reporting raw data for each mistreatment and discrimination item, we assigned a score to each response option (never, 0; once, 1; several times a year, 2; weekly, 3; and several times a week, 4) for each item. To provide a proxy measure of the extent of mistreatment experiences across multiple dimensions, we summed physicians’ responses to each item (including only those who had responded to all the items), with a higher score representing greater exposure to mistreatment and discrimination by patients, families, and visitors (range, 0 to 24). Based on the distribution of total mistreatment scores, we grouped scores into 4 categories: no reported mistreatment and discrimination (score, 0) and scores of 1, 2, or 3 and greater.

We calculated standard descriptive summary statistics and explored associations between variables using the Kruskal-Wallis test or χ^2^ test. All tests were 2-sided with type I error rates of .05. We used multivariable logistic regression to identify factors independently associated with burnout; reporting no mistreatment or discrimination by patients, families, and visitors within the previous year (score of 0); and reporting frequent mistreatment or discrimination by patients, families, and visitors in the previous year (score of ≥3). The multivariable logistic regression model for burnout included demographic and practice characteristics previously shown to be associated with burnout risk^1,2^ and mistreatment and discrimination score categories. The separate multivariable logistic regression models for no mistreatment or discrimination (score of 0) and frequent mistreatment or discrimination (score of ≥3) included demographic and practice characteristics, as previous studies have found associations between these characteristics and mistreatment and discrimination.^6,9,10,13,14,17,18,22,23^ Analyses were conducted in SAS version 9.4 (SAS Institute).

## Results

As previously reported,^20^ survey responders included 1162 of 3671 physicians (31.7%) who received a mailed survey and 6348 of 90 000 physicians (7.1%) who received an electronic survey (overall response rate, 8.0%). Of the 6512 responding physicians, 2450 (37.6%) were female; 369 (7.2%) were Hispanic; 681 (13.3%) were non-Hispanic Asian, Native Hawaiian, or Pacific Islander; 181 (3.5%) were non-Hispanic Black; 182 (3.5%) were Non-Hispanic Indigenous or other; 3633 (70.5%) were non-Hispanic White; and 102 (2.0%) were non-Hispanic 2 or more races. The median age was 54.0 (44.0-62.0) years, physicians worked a median (IQR) 50.0 (40.0-60.0) hours per week and were on call a median (IQR) 1 (0-2) night per week, and most (3571 [56.9%]) worked in private practice. Additional details, including specialty distribution of responders, are shown in eTable 2 in the Supplement. In comparison with all practicing US physicians, our responders to the mailed or electronic survey were slightly older, more likely to be female, and more likely to work in specialties outside of general internal medicine, general pediatrics, family medicine, and obstetrics and gynecology.

Of the 6512 responders, 6249 (96.0%) completed the items on mistreatment and discrimination and were included in the present analysis. The frequency of experiencing each mistreatment and discrimination item at work in the previous year is shown in Table 1. Female physicians were more likely to report experiencing each mistreatment and discrimination behavior in the previous year than male physicians (eTable 3 in the Supplement). Variability in experience by gender and race and ethnicity is shown in Table 2. There were significant differences in the prevalence of reporting experience of each mistreatment and discrimination behavior in the previous year by race and ethnicity, except for being subjected to offensive sexist remarks (eTable 4 in the Supplement).

**Table 1.** Personal Experience of Mistreatment and Discrimination at Work in the Previous Year by Patients, Families, or Visitors

Experience	Respondents, No. (%) (N = 6512)				
	Never	Once	Several times a year	Weekly	Several times a week
Been subjected to					
Racially or ethnically offensive remarks	4446 (70.6)	962 (15.3)	800 (12.7)	56 (0.9)	31 (0.5)
Offensive sexist remarks	4486 (71.3)	782 (12.4)	919 (14.6)	75 (1.2)	34 (0.5)
Unwanted sexual advances	5009 (79.5)	802 (12.7)	457 (7.3)	28 (0.4)	7 (0.1)
Offensive remarks related to sexual orientation	5681 (90.2)	340 (5.4)	240 (3.8)	26 (0.4)	10 (0.2)
Had a patient or his/her family refuse to allow them to provide care due to the physician’s personal attributes	4938 (78.4)	902 (14.3)	427 (6.8)	22 (0.3)	8 (0.1)
Been physically harmed (eg, hit, slapped, kicked)	5372 (85.2)	646 (10.3)	254 (4)	18 (0.3)	12 (0.2)

**Table 2.** Personal Experience of Mistreatment and Bias by Patients, Families, or Visitors Once a Year or More Often by Physician Gender and Race/Ethnicity

Experience	Respondents, No. (%)											
	Male						Female					
	Non-Hispanic					Hispanic or Latino (n = 212)	Non-Hispanic					Hispanic or Latino (n = 157)
	White (n = 2307)	Black or AA (n = 70)	AAPI (n = 354)	Indigenous or other (n = 113)	≥2 Races (n = 44)		White (n = 1315)	Black or AA (n = 111)	AAPI (n = 327)	Indigenous or other (n = 69)	≥2 Races (n = 56)	
Been subjected to												
Offensive racially or ethnically remarks	476 (20.7)	40 (57.1)	182 (51.7)	51 (45.1)	18 (41.9)	69 (32.5)	321 (24.4)	61 (55.0)	192 (59.3)	44 (63.8)	28 (50.0)	73 (46.5)
Offensive sexist remarks	361 (15.7)	7 (10.1)	53 (15.1)	15 (13.3)	11 (25.0)	30 (14.2)	716 (54.6)	49 (44.5)	155 (47.7)	39 (56.5)	30 (53.6)	78 (49.7)
Unwanted sexual advances	363 (15.8)	16 (22.9)	39 (11.1)	21 (18.6)	12 (27.3)	42 (19.8)	422 (32.1)	38 (34.5)	87 (26.7)	21 (30.4)	22 (39.3)	40 (25.5)
Offensive remarks related to sexual orientation	197 (8.6)	4 (5.7)	22 (6.3)	9 (8.0)	9 (20.9)	20 (9.4)	173 (13.2)	11 (9.9)	38 (11.7)	16 (23.2)	9 (16.4)	25 (15.9)
Had a patient or his/her family refuse to allow them to provide care because of the physician’s personal attributes	337 (14.7)	31 (44.3)	114 (32.4)	28 (24.8)	16 (37.2)	47 (22.2)	312 (23.8)	42 (37.8)	117 (36.1)	30 (43.5)	18 (32.1)	55 (35.0)
Been physically harmed (eg, hit, slapped, kicked)	348 (15.1)	6 (8.6)	29 (8.2)	18 (15.9)	14 (31.8)	26 (12.3)	247 (18.8)	12 (10.9)	55 (16.8)	11 (15.9)	8 (14.3)	23 (14.6)

Abbreviations: AA, African American; AAPI, Asian, Native Hawaiian, or Pacific Islander.

Overall, 29.4% of all responders (1849 physicians) had been subjected to racially or ethnically offensive remarks by patients, their families, or visitors once or more within the past year. A higher prevalence of such mistreatment was reported by female physicians than male physicians (826 [34.7%] vs 1014 [26.0%]; *P* < .001) (eTable 3 in the Supplement) and racial and ethnic minority physicians (eTable 4 in the Supplement). More than half of non-Hispanic Black physicians (101 [55.8%]), non-Hispanic Asian, Native Hawaiian, or Pacific Islander physicians (375 [55.4%]), and non-Hispanic Indigenous or other physicians (96 [52.5%]) had experienced offensive racial or ethnic remarks by patients, their family members, or visitors once or more within the past year, while less than one-quarter of White physicians (797 [22.0%]; *P* < .001) had such experiences. Within most racial and ethnic groups, more female than male physicians reported such experiences. For example, 44 of 69 non-Hispanic Indigenous or other female physicians (63.8%) had been subjected to offensive racially or ethnically remarks from patients, families, and visitors in the previous year.

Offensive sexist remarks or unwanted sexual advances by patients, families, or visitors at least once in the previous year were reported by 1810 physicians (28.7%) and 1291 physicians (20.5%), respectively. Such experiences were more frequent for female physicians than male physicians (sexist remarks: 1213 [51.0%] vs 587 [15.1%]; *P* < .001; unwanted sexual advances: 704 [29.6%] vs 585 [15.0%]; *P* < .001) (eTable 3 in the Supplement).

Approximately 1 in 5 physicians (1359 [21.6%]) had experienced a patient or their family refusing to allow them to provide care because of the physician’s personal attributes at least once in the previous year. Overall, 649 non-Hispanic White physicians (17.9%) had experienced a patient or his/her family refusing care due to the physician’s personal attributes, but female physicians and male and female physicians from multiple ethnic and racial groups experienced this much more frequently. A total of 655 female physicians (27.5%) had a patient or his/her family refuse care, and more than 40% of non-Hispanic Black male physicians and non-Hispanic Indigenous female physicians reported a patient or their family refusing to allow them to provide care at least once in the previous year.

Nearly 15% of physicians (930 [14.8%]) reported being physically harmed by patients, their family members, or visitors at least once in the previous year. Nearly a third of non-Hispanic male physicians of 2 or more races (14 [31.8%]) reported physical harm, almost twice that of other groups, although the number of physicians in each category was small.

Fewer physicians (616 [9.8%]) reported being subjected to offensive remarks related to their sexual orientation by patients, families, and visitors at least once in the previous year. Across gender and racial and ethnic groups, more than 1 in 5 non-Hispanic Indigenous or other female physicians (16 [23.2%]) and non-Hispanic female physicians identifying as 2 or more races (9 [20.9%]) reported such experiences.

### Mistreatment and Discrimination Summative Scores

The distribution of mistreatment and discrimination summative scores by gender and race and ethnicity are shown in eFigures 1 and 2 in the Supplement, respectively. Female physicians (eTable 5 in the Supplement) and racial and ethnic minority physicians (eTable 6 in the Supplement) had higher summative mistreatment and discrimination scores. Intersectional analysis between gender and race and ethnicity is shown in eTable 7 in the Supplement. On multivariable analysis, female physicians (vs male physicians) and racial and ethnic minority physicians (vs non-Hispanic White physicians) were more likely to experience frequent mistreatment (female physicians: odds ratio [OR], 2.33; 95% CI, 2.02-2.69; Hispanic: OR, 1.34; 95% CI, 1.04-1.73; non-Hispanic 2 or more races: OR, 1.58; 95% CI, 1.01-2.49; non-Hispanic Asian, Native Hawaiian, or Pacific Islander: OR, 1.33; 95% CI, 1.09-1.61; non-Hispanic Black: OR, 1.59; 95% CI, 1.13-2.23; non-Hispanic Indigenous or other: OR, 1.80; 95% CI, 1.29-2.52) (Table 3). Female physicians had lower odds of not experiencing mistreatment or discrimination after controlling for age, relationship status, specialty, and practice setting (vs male physicians: OR, 0.47; 95% CI, 0.41-0.54). Non-Hispanic Asian, Native Hawaiian, or Pacific islander physicians, non-Hispanic Black physicians, and non-Hispanic Indigenous or other physicians were also less likely than non-Hispanic White physicians to not experience any mistreatment or discrimination (Table 3). Relative to general internal medicine physicians, physicians in specialties with less direct patient contact (eg, pathology, radiology) were at lower risk for mistreatment (vs general internal medicine, pathology: OR, 0.03; 95% CI, 0.01-0.12; radiology: OR, 0.54; 95% CI, 0.34-0.85) while physicians practicing emergency medicine had higher risk (OR, 3.94; 95% CI, 2.76-5.63).

**Table 3.** Multivariable Analysis of Factors Associated With More Frequent Mistreatment and Discrimination or No Mistreatment and Discrimination

Factor	OR (95% CI)	
	More frequent mistreatment and discrimination^a^	No mistreatment or discrimination^b^
Female (vs male)	2.33 (2.02-2.69)	0.47 (0.41-0.54)
Age (for each year older)	0.97 (0.96-0.98)	1.03 (1.02-1.03)
Race and ethnicity (vs Non-Hispanic White)		
Hispanic or Latino	1.34 (1.04-1.73)	0.85 (0.67-1.08)
Non-Hispanic		
≥2 Races	1.58 (1.01-2.49)	0.65 (0.41-1.04)
Asian, Native Hawaiian, or Pacific Islander	1.33 (1.09-1.61)	0.61 (0.51-0.74)
Black or African American	1.59 (1.13-2.23)	0.60 (0.42-0.85)
Indigenous or other	1.80 (1.29-2.52)	0.65 (0.46-0.90)
Relationship status (vs single)		
Married	0.68 (0.55-0.83)	1.17 (0.96-1.44)
Partnered	1.02 (0.73-1.43)	0.77 (0.55-1.08)
Widowed or widower	1.20 (0.64-2.27)	1.08 (0.60-1.96)
Specialty (vs general internal medicine)		
Anesthesiology	0.68 (0.46-1.01)	1.19 (0.83-1.69)
Dermatology	1.38 (0.87-2.19)	0.76 (0.48-1.20)
Emergency medicine	3.94 (2.76-5.63)	0.21 (0.14-0.32)
Family medicine	1.01 (0.72-1.41)	0.87 (0.63-1.20)
General surgery	0.83 (0.53-1.28)	1.10 (0.74-1.63)
General surgery subspecialty	0.80 (0.57-1.13)	1.17 (0.86-1.59)
Internal medicine subspecialty	0.89 (0.65-1.23)	0.95 (0.70-1.27)
Neurology	0.74 (0.48-1.14)	0.92 (0.63-1.36)
Neurosurgery	0.92 (0.45-1.88)	1.23 (0.66-2.26)
Obstetrics and gynecology	0.63 (0.42-0.94)	1.14 (0.79-1.66)
Ophthalmology	0.83 (0.55-1.24)	1.25 (0.87-1.80)
Orthopedic surgery	0.78 (0.52-1.17)	1.22 (0.86-1.73)
Other	0.93 (0.66-1.32)	0.96 (0.70-1.33)
Otolaryngology	1.18 (0.60-2.33)	0.90 (0.48-1.69)
Pathology	0.03 (0.01-0.12)	17.83 (8.92-35.64)
Pediatric subspecialty	0.72 (0.47-1.09)	1.25 (0.85-1.85)
Pediatrics, general	0.50 (0.34-0.73)	1.30 (0.92-1.84)
Physical medicine and rehabilitation	1.31 (0.81-2.10)	0.86 (0.55-1.37)
Preventative or occupational medicine	0.57 (0.18-1.85)	1.08 (0.42-2.81)
Psychiatry	1.21 (0.87-1.68)	0.85 (0.62-1.16)
Radiation oncology	0.68 (0.29-1.59)	1.61 (0.79-3.30)
Radiology	0.54 (0.34-0.85)	2.23 (1.50-3.33)
Urology	1.31 (0.54-3.19)	0.93 (0.40-2.15)
Practice setting (vs nonacademic private practice or group)		
Academic medical center	0.88 (0.75-1.03)	1.05 (0.91-1.22)
Active military practice	1.07 (0.45-2.53)	0.95 (0.42-2.17)
Other	1.00 (0.81-1.23)	1.00 (0.83-1.21)
Veterans’ hospital	1.03 (0.68-1.57)	0.91 (0.61-1.36)

Abbreviation: OR, odds ratio.

^a^ Respondents with a mistreatment or discrimination summative score of 3 or higher.

^b^ Respondents with a mistreatment or discrimination summative score of 0 were categorized as not experiencing mistreatment or discrimination by patients, families, or visitors.

#### Mistreatment and Discrimination by Patients, Families, and Visitors and Physician Burnout

An exposure-response association was found between frequency of experiencing each mistreatment and discrimination behavior and prevalence of high emotional exhaustion, high depersonalization, overall burnout (Figure). For example, as the frequency of being subjected to racially or ethnically offensive remarks increased from never to several times a week, we observed an increase in the percentage of participants with high emotional exhaustion (28.6% to 67.6%), high depersonalization (19.8% to 56.7%), and burnout (34.5% to 76.7%). As the frequency of individual experiences of mistreatment and discrimination increased from never to several times a week, the emotional exhaustion score increased by an average of 14 points and depersonalization score increased by an average of 8 points. For example, those who had never been subjected to an racially or ethnically offensive remark several times a week had a mean (SD) emotional exhaustion score of 19.8 (13.0) and mean (SD) depersonalization score of 5.4 (5.8), while those who reported such experiences several times a week had a mean (SD) emotional exhaustion score of 34.5 (13.9) and mean (SD) depersonalization score of 12.6 (9.0; both *P* < .001) (eTable 8 in the Supplement). Similarly, as the total mistreatment and discrimination score increased, so did the frequency of symptoms of emotional exhaustion and depersonalization (eFigures 3 and 4 in the Supplement).

**Figure.**
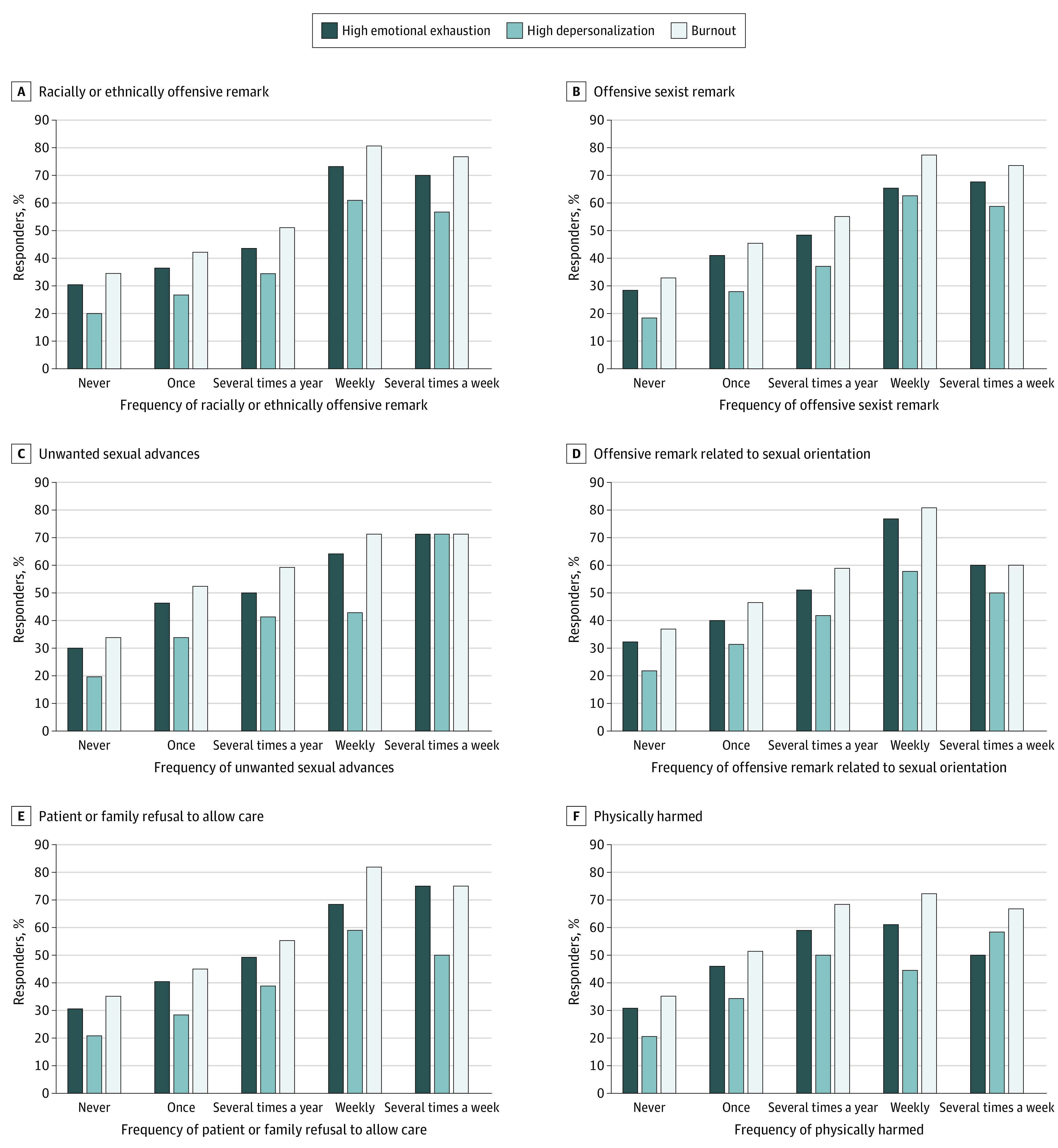
Percentage of Respondents With High Emotional Exhaustion, High Depersonalization, and Burnout, by Frequency of Experiencing Different Forms of Mistreatment and Discrimination As the frequency of the behavior increased, the percentage of participants with high emotional exhaustion, high depersonalization, and burnout significantly increased (all *P* < .001).

Factors independently associated with burnout in multivariable analysis are shown in Table 4. As the summative mistreatment and discrimination by patients, families, or visitors score increased, so did the odds of burnout (referent score of 0; score of 1: OR, 1.27; 95% CI, 1.04-1.55; score of 2: OR, 1.70; 95% CI, 1.38-2.08; score of ≥3: OR, 2.20; 95% CI, 1.89-2.57) independent of age, gender, relationship status, race and ethnicity, specialty, practice setting, work hours, and call frequency. There was no difference in the odds of burnout by gender after controlling for summative mistreatment and discrimination score as well as other personal and professional factors. Differences in the odds of burnout did persist by racial and ethnic group on the multivariable analysis (overall *P* < .001) with non-Hispanic Asian, Native Hawaiian, and Pacific Islander physicians having lower odds of burnout than non-Hispanic White physicians (OR, 0.67; 95% CI, 0.54-0.79; *P* < .001) after controlling for the summative mistreatment and discrimination score and other personal and professional factors.

**Table 4.** Multivariable Analysis of Factors Associated With Burnout

Factor	OR (95% CI)
Mistreatment and discrimination score^a^	
0	1 [Reference]
1	1.27 (1.04-1.55)
2	1.70 (1.38-2.08)
≥3	2.20 (1.89-2.57)
Hours worked per week (for each additional hour)	1.02 (1.01-1.02)
Nights on call per week (for each additional night)	1.05 (1.02-1.08)
Male (vs female)	0.90 (0.78-1.04)
Age (for each year older)	0.97 (0.967-0.979)
Race and ethnicity (vs Non-Hispanic White)	
Hispanic or Latino	0.74 (0.58-0.94)
Non-Hispanic	
≥2 Races	1.27 (0.82-1.96)
Asian, Native Hawaiian, or Pacific Islander	0.67 (0.54-0.79)
Black or African American	0.81 (0.58-1.14)
Indigenous or other	0.73 (0.52-1.03)
Relationship status (vs single)	
Married	0.69 (0.57-0.84)
Partnered	0.85 (0.62-1.18)
Widowed or widower	0.49 (0.25-0.97)
Specialty (vs general internal medicine)	
Anesthesiology	0.78 (0.54-1.12)
Dermatology	0.78 (0.49-1.24)
Emergency medicine	1.59 (1.12-2.28)
Family medicine	1.24 (0.90-1.72)
General surgery	0.74 (0.49-1.11)
General surgery subspecialty	0.46 (0.33-0.64)
Internal medicine subspecialty	0.91 (0.67-1.23)
Neurology	0.89 (0.60-1.32)
Neurosurgery	0.63 (0.33-1.20)
Obstetrics and gynecology	0.69 (0.47-1.00)
Ophthalmology	0.71 (0.48-1.04)
Orthopedic surgery	0.65 (0.45-0.94)
Other	0.69 (0.49-0.95)
Otolaryngology	0.87 (0.45-1.67)
Pathology	0.65 (0.41-1.04)
Pediatric subspecialty	0.55 (0.36-0.84)
Pediatrics, general	0.85 (0.59-1.21)
Physical medicine and rehabilitation	0.78 (0.49-1.24)
Preventative or occupational medicine	0.48 (0.16-1.42)
Psychiatry	0.87 (0.63-1.19)
Radiation oncology	0.73 (0.34-1.55)
Radiology	1.03 (0.70-1.54)
Urology	0.77 (0.33-1.79)
Practice setting (vs nonacademic private practice or group)	
Academic medical center	0.73 (0.62-0.85)
Active military practice	0.74 (0.34-1.61)
Other	0.83 (0.68-1.01)
Veterans’ hospital	0.80 (0.53-1.20)

Abbreviation: OR, odds ratio.

^a^ Responses to the mistreatment and discrimination items were summed, with a higher score representing greater exposure to mistreatment and discrimination by patients, families, and visitors (range 0 to 24).

## Discussion

In this national sample of US physicians, 20% to 30% reported experiencing racially or ethnically offensive remarks, offensive sexist remarks, or unwanted sexual advances by patients, families, and visitors. In addition, 21.6% experienced a patient or their family refusing to allow them to provide care because of the physician’s personal attributes at least once in the previous year. Approximately 10% to 15% had been subjected to offensive remarks related to their sexual orientation or been physically harmed by patients, families, or visitors at least once in the previous year. Female gender and minority race and ethnicity were associated with higher risk of experiencing mistreatment and discrimination by patients, families, and visitors independent of specialty, practice settings, and other professional characteristics. Physicians who were mistreated or discriminated against by patients, families, or visitors were more likely to have symptoms of burnout, a finding that persisted on multivariable analysis controlling for other personal and professional characteristics.

The risk of burnout increased by 27% to 120% as the summative mistreatment and discrimination experience score increased. As frequency of individual experiences of mistreatment and discrimination increased from never to several times a week, the emotional exhaustion score increased by an average of 14 points and the depersonalization score increased by an average of 8 points. Previous research supports that each 1-point increase in emotional exhaustion is associated with an 11% higher odds of a physician believing they have recently committed a major medical error and recently having experienced suicidal ideation.^24,25,26^Additionally, each 1-point increase in depersonalization is associated with 5% higher odds of a physician believing they have recently committed a major medical error and 7% higher odds of a physician recently having experienced suicidal ideation.^24,25,26^ This suggests that the impact of experiencing mistreatment and discrimination by patients, families, and visitors on physicians observed in this study is likely to be clinically significant.

Previous studies have suggested a higher prevalence of burnout among female physicians relative to male physicians.^27,28^ The results of this national study indicate that, once experiences of mistreatment and discrimination by patients, families, and visitors and other factors (age, race and ethnicity, relationship status, specialty, practice setting) are considered, female physicians were not at higher risk for burnout than male physicians. Having such negative experiences was independently associated with burnout in both female and male physicians, but female physicians were more likely to experience each domain of mistreatment and discrimination by patients, families, and visitors. These findings are consistent with a large, longitudinal multispecialty study of US resident physicians in which differences in the prevalence of burnout between the genders were, at least in part, because of differing levels of exposure to negative interactions with other health care workers (attending physicians, nursing staff, clerical and administrative staff, and other health care workers) and patients for women vs men.^29^

In this study, female and racial and ethnic minority physicians reported experiencing mistreatment and discrimination from patients, families, and visitors more frequently than non-Hispanic White male physicians, a finding that remained statistically significant on multivariable analysis. Nearly two-thirds (64%) of non-Hispanic Indigenous female physicians had been subjected to racially or ethnically offensive remarks from patients, families, and visitors in the previous year, and nearly half of female physicians across all ethnic and racial groups had been subjected to offensive sexist remarks. While only 14.7% of non-Hispanic White physicians had experienced a patient or his/her family refusing care due to the physician’s personal attributes, the prevalence was twice that—or more—among female physicians and male and female physicians from multiple ethnic and racial groups. Given the association between mistreatment experiences and burnout, strategies to improve the work lives and retention of minoritized and marginalized physicians should include a focus on policies and procedures that promote an equitable and inclusive work environment.

Physical violence by patients, families, or visitors was reported by nearly 15% of physicians in this cohort, which included a range of specialties and practice settings. A recent systematic review and meta-analysis reported that 24.4% of health care workers globally and 37.3% in North America experienced physical violence within the previous year, with rates varying by gender, age, type of health care worker, and work location.^22^ In 2019, there were approximately 13 000 reported cases of nonfatal occupational intentional injuries to health care workers by other persons in health care settings.^30^ Most were experienced by women, and on average, 20% resulted in 3 to 5 days away from work to recover. Experience of workplace violence has been associated with depressive symptoms, burnout, job dissatisfaction, and turnover in studies of non-US physicians and nurses.^31,32,33,34,35,36,37,38^ The Occupational Safety and Health Administration and Joint Commission have guidelines or policy requirements regarding workplace violence.^39^ Unfortunately, little is known about how best to mitigate risk of workplace violence or other forms of mistreatment and discrimination by patients, families, and visitors.^10^

Findings from this study suggest that organizational efforts to mitigate the risk of burnout among physicians should include strategies that appropriately deal with and reduce mistreatment and discrimination by patients, families, and visitors. The study highlights an important focal point to improve the practice environment for chief wellness officers, chief medical officers, quality officers, and other institutional leaders designated to address system-level factors causing high levels of workplace stress for physicians and other members of the health care team. The results also highlight a dimension for attention by chief diversity officers, security leaders, operational leaders, patient experience officers, and workplace violence leaders to promote an environment of safety, equity, inclusion, and belonging. A coordinated strategy involving operational; security; diversity, equity, and inclusion; and well-being leaders that prioritizes this domain, builds a coalition, leverages existing resources, and addresses gaps may accelerate effective change to improve the work environment.

### Limitations

This study has several limitations. We explored a limited number of mistreatment and discrimination behaviors and explored differences only by gender, race, and ethnicity when there are multiple other intersecting identities, visible and not. The survey asked responders to recall experiences of mistreatment and discrimination over the past year, and as a retrospective survey, the data are subject to recall and selection bias. We explored associations between the frequency of experiencing each form of mistreatment and discrimination behaviors arising from patients, family members, and visitors as well as a proxy measure of aggregate mistreatment and discrimination experiences. Although this measure provides some insights into the impact of mistreatment experiences holistically, it has limitations. For example, it is unknown how the experience of multiple types of mistreatment and discrimination impacts an individual relative to experiencing a single type of mistreatment and discrimination more frequently. Since it is cross-sectional, the current study also cannot determine causation or the potential direction of the associations observed. However, previous longitudinal studies of medical students and residents have demonstrated that mistreatment experiences (primarily originating from coworkers) predicted subsequent burnout 1 year later after controlling for baseline measures of burnout and other personal and professional factors.^29,40^ Although the sample was obtained from a nearly complete record of US physicians and the secondary survey of nonresponders suggests participants are broadly representative of US physicians with respect to demographic characteristics and burnout scores, response bias remains a concern and may influence prevalence estimates of mistreatment by patients, families, and visitors.

## Conclusions

In this national study, practicing physicians commonly experienced mistreatment and discrimination by patients and their families and visitors, with female physicians and racial and ethnic minority physicians at particular risk. Physicians with such mistreatment and discrimination experiences were more likely to have symptoms of burnout. After taking experiences of mistreatment and discrimination by patients, families, and visitors and other factors (age, race, ethnicity, relationship status, specialty, practice setting) into account, female physicians were not at higher risk for burnout than male physicians, suggesting previously reported gender differences in burnout rates may at least partially be because of differences in experiences of mistreatment at work. Effective strategies are needed to reduce the frequency of inappropriate patient, family, and visitor behaviors as well as appropriately address them when they occur.

## Notes

Supplement. **eAppendix.** Additional Details on Sampling **eTable 1.** Survey Items Related to Mistreatment and Discrimination by Patients, Family Members, and Visitors **eTable 2.** Demographic Characteristics of the 6512 US Physicians **eTable 3.** Experience of Mistreatment Once or More Within the Past Year by Gender **eTable 4.** Experience of Mistreatment Once or More Within the Past Year by Race and Ethnicity Among Physicians **eTable 5.** Mistreatment and Discrimination Score Category by Gender **eTable 6.** Mistreatment and Discrimination Score Category by Race and Ethnicity **eTable 7.** Mistreatment and Discrimination Score by Gender and Race and Ethnicity **eTable 8.** Personal Experience of Mistreatment and Experience of Discrimination in the Previous Year Among Physicians and Mean Emotional Exhaustion and Depersonalization Scores **eFigure 1.** Mistreatment and Discrimination Score Distribution by Gender **eFigure 2.** Mistreatment and Discrimination Score Distribution by Race and Ethnicity **eFigure 3.** Mistreatment and Discrimination Score and Mean Emotional Exhaustion and Depersonalization Scores **eFigure 4.** Mistreatment and Discrimination Score and Burnout
