# Supplementary material for: Physicians’ Experiences With Mistreatment and Discrimination by Patients, Families, and Visitors and Association With Burnout
Source: JAMA Netw Open. 2022 May 19;5(5):e2213080. doi: 10.1001/jamanetworkopen.2022.13080 (PMC9121189; doi:10.1001/jamanetworkopen.2022.13080)
Supplement: Supplement. — eAppendix. Additional Details on Sampling eTable 1. Survey Items Related to Mistreatment and Discrimination by Patients, Family Members, and Visitors eTable 2. Demographic Characteristics of the 6512 US Physicians eTable 3. Experience of Mistreatment Once or More Within the Past Year by Gender eTable 4. Experience of Mistreatment Once or More Within the Past Year by Race and Ethnicity Among Physicians eTable 5. Mistreatment and Discrimination Score Category by Gender eTable 6. Mistreatment and Discrimination Score Category by Race and Ethnicity eTable 7. Mistreatment and Discrimination Score by Gender and Race and Ethnicity eTable 8. Personal Experience of Mistreatment and Experience of Discrimination in the Previous Year Among Physicians and Mean Emotional Exhaustion and Depersonalization Scores eFigure 1. Mistreatment and Discrimination Score Distribution by Gender eFigure 2. Mistreatment and Discrimination Score Distribution by Race and Ethnicity eFigure 3. Mistreatment and Discrimination Score and Mean Emotional Exhaustion and Depersonalization Scores eFigure 4. Mistreatment and Discrimination Score and Burnout [file jamanetwopen-e2213080-s001.pdf]

Supplemental Online Content

Dyrbye LN, West CP, Sinsky CA, et al. Physicians' experiences with mistreatment and discrimination by patients, families, and visitors and association with burnout. *JAMA Netw Open.* 2022;5(5):e2213080. doi:10.1001/jamanetworkopen.2022.13080

eAppendix. Additional Details on Sampling

eTable 1. Survey Items Related to Mistreatment and Discrimination by Patients, Family Members, and Visitors

eTable 2. Demographic Characteristics of the 6512 US Physicians

eTable 3. Experience of Mistreatment Once or More Within the Past Year by Gender

eTable 4. Experience of Mistreatment Once or More Within the Past Year by Race and Ethnicity Among Physicians

eTable 5. Mistreatment and Discrimination Score Category by Gender

eTable 6. Mistreatment and Discrimination Score Category by Race and Ethnicity

eTable 7. Mistreatment and Discrimination Score by Gender and Race and Ethnicity

eTable 8. Personal Experience of Mistreatment and Experience of Discrimination in the Previous Year Among Physicians and Mean Emotional Exhaustion and Depersonalization Scores

eFigure 1. Mistreatment and Discrimination Score Distribution by Gender

eFigure 2. Mistreatment and Discrimination Score Distribution by Race and Ethnicity

eFigure 3. Mistreatment and Discrimination Score and Mean Emotional Exhaustion and Depersonalization Scores

eFigure 4. Mistreatment and Discrimination Score and Burnout

This supplemental material has been provided by the authors to give readers additional information about their work.

© 2022 Dyrbye LN et al. *JAMA Network Open.*

eAppendix. Additional Details on Sampling

The sample was randomly selected from the American Medical Association Physician Masterfile, a nearly complete record of all US physicians independent of American Medical Association membership, and comprised of practicing physicians in all specialties. Medical students and residents were excluded from the sample selection. We oversampled physicians in fields other than general internal medicine, general pediatrics, family medicine, and obstetrics/gynecology to increase the sample of physicians from smaller specialties. Sample specialty distribution was constructed to match that of the 2014 and 2017 sample compositions and is shown below.

	2020 Sample			
Specialty	Paper Mailing		Electronic Survey	
Anesthesiology	170	4.3%	4042	4.3%
Dermatology	87	2.2%	2056	2.2%
Emergency Medicine	194	4.8%	4600	4.8%
Family Medicine	340	8.5%	8074	8.5%
Internal Medicine - General	360	9.0%	8553	9.0%
Internal Medicine - Subspecialty	527	13.2%	12507	13.2%
Neurology	128	3.2%	3050	3.2%
Obstetrics & Gynecology	173	4.3%	4099	4.3%
Ophthalmology	129	3.2%	3059	3.2%
Other	129	3.2%	3057	3.2%
Pathology	86	2.1%	2034	2.1%
Pediatrics - General	254	6.4%	6035	6.4%
Pediatrics - Subspecialty	120	3.0%	2859	3.0%
Physical Medicine	79	2.0%	1877	2.0%
Psychiatry	254	6.4%	6034	6.4%
Radiology	213	5.3%	5062	5.3%
Surgery - General	206	5.1%	4880	5.1%
Surgery - Subspecialty	552	13.8%	13100	13.8%

© 2022 Dyrbye LN et al. *JAMA Network Open.*

eTable 1. Survey Items Related to Mistreatment and Discrimination by Patients, Family Members, and Visitors

For each of the following behaviors, please indicate the frequency you personally experienced that behavior at work within the previous year. Include in your response any behaviors performed by patients, families, or visitors. Do not include behaviors of faculty, nurses, residents/interns, other institution employees or staff.

In the last 12 months, how frequently have you

Been subjected to racially or ethnically offensive remarks at work by a patient, family member, or patient visitor?
Been subjected to offensive sexist remarks at work by a patient, family member, or patient visitor?
Been subjected to unwanted sexual advances at work by a patient, family member, or patient visitor?
Been subjected to offensive remarks related to sexual orientation at work by a patient, family member, or patient visitor?
Had a patient or his/her family refuse to allow me to provide care for the patient due to my personal attributes (gender, race/ethnicity, sexual orientation, or other)?
Been physically harmed (e.g., hit, slapped, kicked) at work by a patient, family member, or patient visitor?

Response options: Never, once, several times a year, weekly, several times a week

© 2022 Dyrbye LN et al. *JAMA Network Open.*

eTable 2. Demographic Characteristics of the 6512 US Physicians

	All (n = 6512)	Female (n = 2450)	Male (n = 4062)
**Race / Ethnicity**			
Non-Hispanic White	3622 (70.5%)	1315 (64.6%)	2307 (74.4%)
Non-Hispanic Black/AA	181 (3.5%)	111 (5.5%)	70 (2.3%)
Non-Hispanic AAPI	681 (13.3%)	327 (16.1%)	354 (11.4%)
Non-Hispanic Indigenous/other	182 (3.5%)	69 (3.4%)	113 (3.6%)
Non-Hispanic 2 or more races	102 (2.0%)	57 (2.8%)	45 (1.5%)
Hispanic/Latino/a	369 (7.2%)	157 (7.7%)	212 (6.8%)
Missing	1375	414	961
**Age in Years**			
Median	54.0	49.0	58.0
<35	225 (3.6%)	135 (5.7%)	90 (2.3%)
35-44	1363 (21.7%)	714 (30.0%)	649 (16.6%)
45-54	1647 (26.2%)	744 (31.2%)	903 (23.1%)
55-64	1856 (29.5%)	586 (24.6%)	1270 (32.5%)
≥65	1203 (19.1%)	203 (8.5%)	1000 (25.6%)
missing	218	68	150
**Specialty**			
Anesthesiology	287 (4.6%)	84 (3.5%)	203 (5.2%)
Dermatology	155 (2.5%)	83 (3.5%)	72 (1.9%)
Emergency Medicine	343 (5.5%)	122 (5.1%)	221 (5.7%)
Family Medicine	435 (7.0%)	199 (8.4%)	236 (6.1%)
General Surgery	210 (3.4%)	45 (1.9%)	165 (4.3%)
General Surgery Subspecialty	490 (7.8%)	132 (5.6%)	358 (9.2%)
Internal Medicine Subspecialty	434 (6.9%)	200 (8.4%)	234 (6.0%)
Neurology	603 (9.7%)	191 (8.0%)	412 (10.6%)
Neurosurgery	222 (3.6%)	79 (3.3%)	143 (3.7%)
Obstetrics and gynecology	64 (1.0%)	9 (0.4%)	55 (1.4%)
Ophthalmology	258 (4.1%)	172 (7.2%)	86 (2.2%)
Orthopedic Surgery	264 (4.2%)	68 (2.9%)	196 (5.1%)
Other	313 (5.0%)	31 (1.3%)	282 (7.3%)
Otolaryngology	57 (0.9%)	10 (0.4%)	47 (1.2%)
Pathology	395 (6.3%)	136 (5.7%)	259 (6.7%)
Pediatric Subspecialty	161 (2.6%)	74 (3.1%)	87 (2.2%)
Pediatrics-General	325 (5.2%)	217 (9.1%)	108 (2.8%)
Physical Medicine and Rehabilitation	224 (3.6%)	127 (5.3%)	97 (2.5%)
Preventative/Occupational Medicine	146 (2.3%)	57 (2.4%)	89 (2.3%)
Psychiatry	24 (0.4%)	6 (0.3%)	18 (0.5%)
Radiation Oncology	505 (8.1%)	236 (9.9%)	269 (6.9%)

© 2022 Dyrbye LN et al. *JAMA Network Open.*

Radiology	52 (0.8%)	14 (0.6%)	38 (1.0%)
Urology	243 (3.9%)	73 (3.1%)	170 (4.4%)
Urology	38 (0.6%)	9 (0.4%)	29 (0.7%)
Missing	264	76	188
**Hours Worked Per Week**			
Median (IQR)	50.0 (40.0, 60.0)	48.0 (40.0, 60.0)	50.0 (40.0, 60.0)
<40 hrs	1255 (19.4%)	564 (23.3%)	691 (17.1%)
40-49 hrs	1535 (23.7%)	662 (27.3%)	873 (21.6%)
50-59 hrs	1561 (24.1%)	542 (22.4%)	1019 (25.2%)
60-69 hrs	1358 (21.0%)	442 (18.2%)	916 (22.7%)
70-79 hrs	359 (5.6%)	97 (4.0%)	262 (6.5%)
≥80 hrs	400 (6.2%)	118 (4.9%)	282 (7.0%)
Missing	44	25	19
**Number of Nights on Call Per Week**			
Median (IQR)	1.0 (0.0, 2.0)	1.0 (0.0, 2.0)	1.0 (0.0, 3.0)
**Primary Practice Setting**			
Private practice	3570 (56.9%)	1193 (50.4%)	2377 (60.9%)
Academic Medical Center	1760 (28.1%)	758 (32.0%)	1002 (25.7%)
Veterans' hospital	140 (2.2%)	60 (2.5%)	80 (2.1%)
Active military practice	36 (0.6%)	13 (0.5%)	23 (0.6%)
Other	763 (12.2%)	345 (14.6%)	418 (10.7%)
Missing	243	81	162

© 2022 Dyrbye LN et al. *JAMA Network Open.*

eTable 3. Experience Mistreatment Once or More Within the Past Year by Gender

	Male (n=4062) N (%)	Female (n=2450) N (%)	p-value N (%)
Been subjected to racially or ethnically offensive remarks	1014 (26.0%)	826 (34.7%)	<0.001
Been subjected to offensive sexist remarks	587 (15.1%)	1213 (51.0%)	<0.001
Been subjected to unwanted sexual advances	585 (15.0%)	704 (29.6%)	<0.001
Been subjected to offensive remarks related to sexual orientation	314 (8.1%)	298 (12.5%)	<0.001
Had a patient or his/her family refuse to allow them to provide care due to the physician's personal attributes	699 (17.9%)	655 (27.5%)	<0.001
Been physically harmed (e.g., hit, slapped, kicked)	527 (13.5%)	398 (16.7%)	0.001

© 2022 Dyrbye LN et al. *JAMA Network Open.*

eTable 4. Experience Mistreatment Once or More Within the Past Year by Race and Ethnicity Among Physicians

	Non-Hispanic White (n=3622) No. (%)	Non-Hispanic Black/AA (n=181) No. (%)	Non-Hispanic AAPI (n=681) No. (%)	Non-Hispanic Indigenous/other (n=182) No. (%)	Non-Hispanic 2 or more races (n=102) No. (%)	Hispanic/ Latino/a (n =369) No. (%)	p-value
Been subjected to offensive racially or ethnically remarks	797 (22.0%)	101 (55.8%)	375 (55.4%)	96 (52.5%)	47 (47.0%)	142 (38.5%)	<0.001
Been subjected to offensive sexist remarks	1078 (29.8%)	56 (31.3%)	209 (30.9%)	55 (30.1%)	41 (40.6%)	108 (29.3%)	0.32
Been subjected to unwanted sexual advances	786 (21.7%)	54 (30.0%)	126 (18.6%)	43 (23.5%)	34 (33.7%)	82 (22.2%)	0.001
Been subjected to offensive remarks related to sexual orientation	370 (10.2%)	15 (8.3%)	60 (8.8%)	26 (14.3%)	19 (19.2%)	45 (12.2%)	0.01
Had a patient or his/her family refuse to allow them to provide care due to the physician's personal attributes	649 (17.9%)	73 (40.3%)	231 (34.1%)	59 (32.2%)	35 (35.0%)	102 (27.6%)	<0.001
Been physically harmed (e.g., hit, slapped, kicked)	597 (16.5%)	18 (10.0%)	84 (12.4%)	29 (15.8%)	22 (21.8%)	49 (13.3%)	0.01

© 2022 Dyrbye LN et al. *JAMA Network Open.*

eTable 5. Mistreatment and Discrimination Score by Gender

Mistreatment or Discrimination Score [1]	Overall N (%)	Male N (%)	Female N (%)	p-value
0	3008 (48.1%)	2191 (56.4%)	817 (34.5%)	<0.001
1	790 (12.6%)	516 (13.3%)	274 (11.6%)	
2	704 (11.3%)	388 (10.0%)	316 (13.4%)	
3+	1747 (28.0%)	787 (20.3%)	960 (40.6%)	

[1] Responses to the mistreatment and discrimination items were summed with a higher score representing greater exposure to mistreatment and discrimination by patients, families, and visitors (range 0 to 24)

© 2022 Dyrbye LN et al. *JAMA Network Open.*

eTable 6. Mistreatment and Discrimination Score by Race and Ethnicity

Mistreatment or Discrimination Score [1]	Non-Hispanic White No. (%)	Non-Hispanic Black/AA No. (%)	Non-Hispanic AAPI No. (%)	Non-Hispanic Indigenous/other No. (%)	Non-Hispanic 2 or more races No. (%)	Hispanic/Latino No. (%)	p-value
0	1819 (50.5%)	56 (31.6%)	236 (35.1%)	71 (39.0%)	32 (33.0%)	166 (45.0%)	<0.0001
1	455 (12.6%)	20 (11.3%)	99 (14.7%)	26 (14.3%)	13 (13.4%)	37 (10.0%)	
2	379 (10.5%)	24 (13.6%)	95 (14.1%)	15 (8.2%)	10 (10.3%)	44 (11.9%)	
≥3	952 (26.4%)	77 (43.5%)	243 (36.1%)	70 (38.5%)	42 (43.3%)	122 (33.1%)	

[1] Responses to the mistreatment and discrimination items were summed with a higher score representing greater exposure to mistreatment and discrimination by patients, families, and visitors (range 0 to 24)

© 2022 Dyrbye LN et al. *JAMA Network Open.*

eTable 7. Mistreatment and Discrimination Score by Gender and Race and Ethnicity

Mistreatment or Discrimination Score [1]	Non-Hispanic White No. (%)		Non-Hispanic Black/AA No. (%)		Non-Hispanic AAPI No. (%)		Non-Hispanic Indigenous/other No. (%)		Non-Hispanic 2 or more races No. (%)		Hispanic/Latino No. (%)	
	Female	Male	Female	Male	Female	Male	Female	Male	Female	Male	Female	Male
0	468 (35.7%)	1349 (58.9%)	31 (28.7%)	25 (36.2%)	93 (29.0%)	143 (40.7%)	15 (21.7%)	56 (50.0%)	15 (27.3%)	17 (41.5%)	55 (35.0%)	111 (52.4%)
1	148 (11.3%)	306 (13.4%)	15 (13.9%)	5 (7.2%)	38 (11.8%)	61 (17.4%)	12 (17.4%)	14 (12.5%)	7 (12.7%)	6 (14.6%)	10 (6.4%)	27 (12.7%)
2	170 (13.0%)	209 (9.1%)	14 (13.0%)	10 (14.5%)	46 (14.3%)	49 (14.0%)	6 (8.7%)	9 (8.0%)	9 (16.4%)	1 (2.4%)	21 (13.4%)	23 (10.8%)
≥3	524 (40.0%)	427 (18.6%)	48 (44.4%)	29 (42.0%)	144 (44.9%)	98 (27.9%)	36 (52.2%)	33 (29.5%)	24 (43.6%)	17 (41.5%)	71 (45.2%)	51 (24.1%)

[1] Responses to the mistreatment and discrimination items were summed with a higher score representing greater exposure to mistreatment and discrimination by patients, families, and visitors (range 0 to 24)

© 2022 Dyrbye LN et al. *JAMA Network Open.*

eTable 8. Personal Experience of Mistreatment and Experience of Discrimination in the Previous Year Among Physicians and Mean Emotional Exhaustion and Depersonalization Scores

	Mean EE	P value	Mean DP	P-value
Been subjected to offensive racially or ethnically remarks		<0.0001		<0.0001
Several times a week	34.5 (13.92)		12.6 (8.98)	
Weekly	34.6 (13.88)		12.8 (7.90)	
Several times a year	24.8 (12.77)		8.1 (6.74)	
Once	22.3 (12.61)		6.9 (6.24)	
Never	19.8 (13.02)		5.4 (5.76)	
Been subjected to offensive sexist remarks		<0.0001		<0.0001
Several times a week	34.3 (13.44)		13.4 (8.61)	
Weekly	31.3 (12.69)		12.2 (7.41)	
Several times a year	26.4 (12.61)		8.7 (6.71)	
Once	23.6 (12.47)		7.0 (6.00)	
Never	19.2 (12.87)		5.2 (5.71)	
Been subjected to unwanted sexual advances		<0.0001		<0.0001
Several times a week	34.0 (14.89)		15.4 (10.37)	
Weekly	29.1 (12.58)		10.2 (6.88)	
Several times a year	27.5 (12.75)		9.6 (7.11)	
Once	24.8 (12.87)		7.9 (6.52)	
Never	19.8 (12.92)		5.4 (5.78)	
Been subjected to offensive remarks related to sexual orientation		<0.0001		<0.0001
Several times a week	32.6 (15.86)		13.1 (9.65)	
Weekly	33.5 (13.23)		11.6 (7.16)	
Several times a year	26.9 (13.25)		9.7 (7.43)	
Once	23.2 (13.05)		7.5 (6.38)	
Never	20.6 (13.04)		5.8 (5.97)	
Had a patient or his/her family refuse to allow them to provide care due to the physician's personal attributes		<0.0001		<0.0001
Several times a week	35.4 (14.71)		12.3 (10.15)	
Weekly	31.5 (15.38)		11.5 (7.90)	
Several times a year	25.6 (13.58)		9.1 (7.15)	
Once	23.4 (13.20)		7.2 (6.74)	
Never	20.2 (12.92)		5.6 (5.78)	
Been physically harmed (e.g., hit, slapped, kicked)		<0.0001		<0.0001
Several times a week	33.1 (16.18)		13.3 (10.06)	
Weekly	29.7 (12.94)		10.9 (7.65)	
Several times a year	28.6 (12.22)		10.8 (7.26)	
Once	25.5 (13.23)		8.2 (6.81)	
Never	20.1 (12.93)		5.6 (5.81)	

© 2022 Dyrbye LN et al. *JAMA Network Open.*

eFigure 1. Mistreatment and Discrimination Score Distribution by Gender

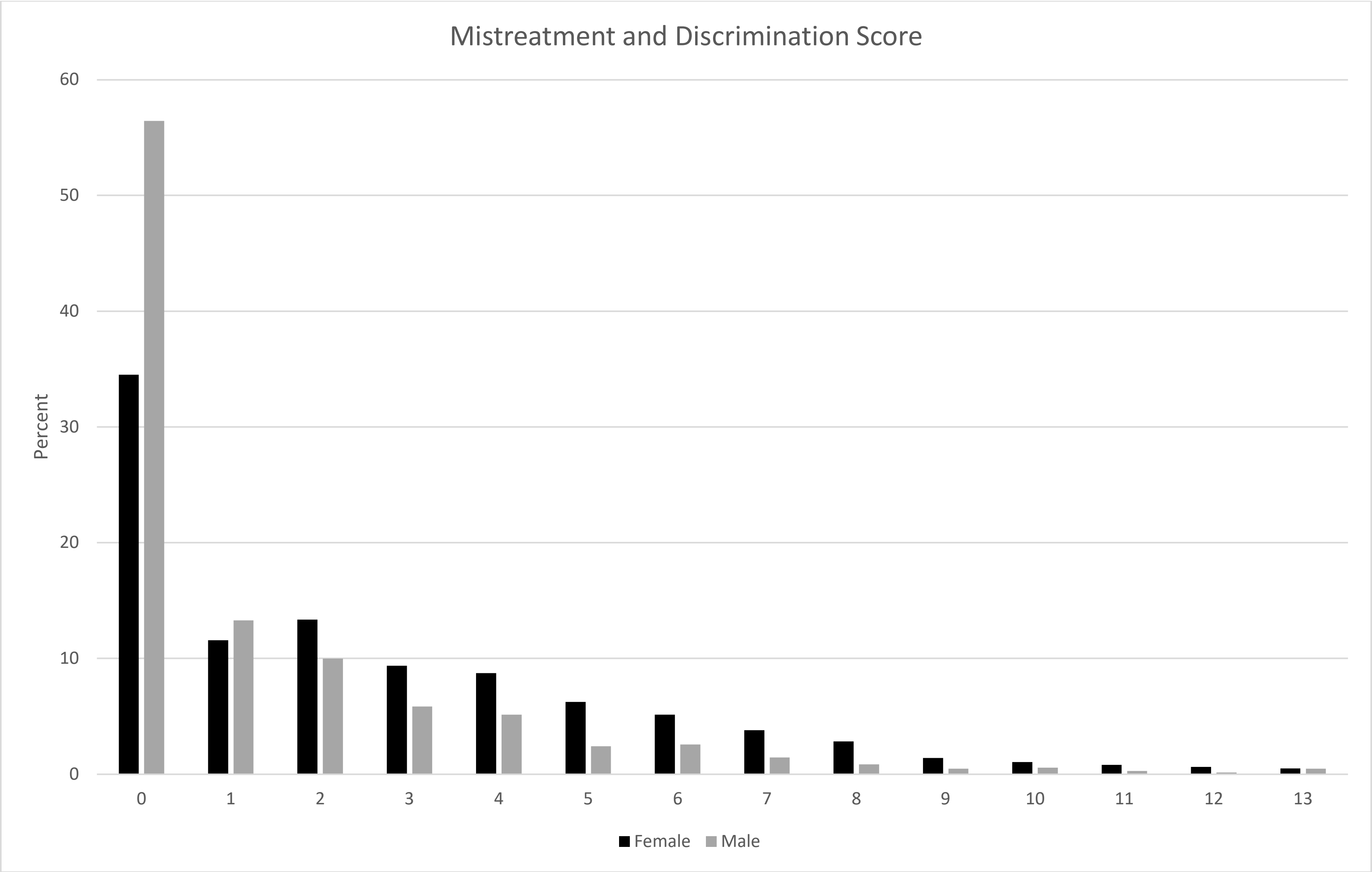

© 2022 Dyrbye LN et al. *JAMA Network Open.*

eFigure 2. Mistreatment and Discrimination Score Distribution by Race/Ethnicity

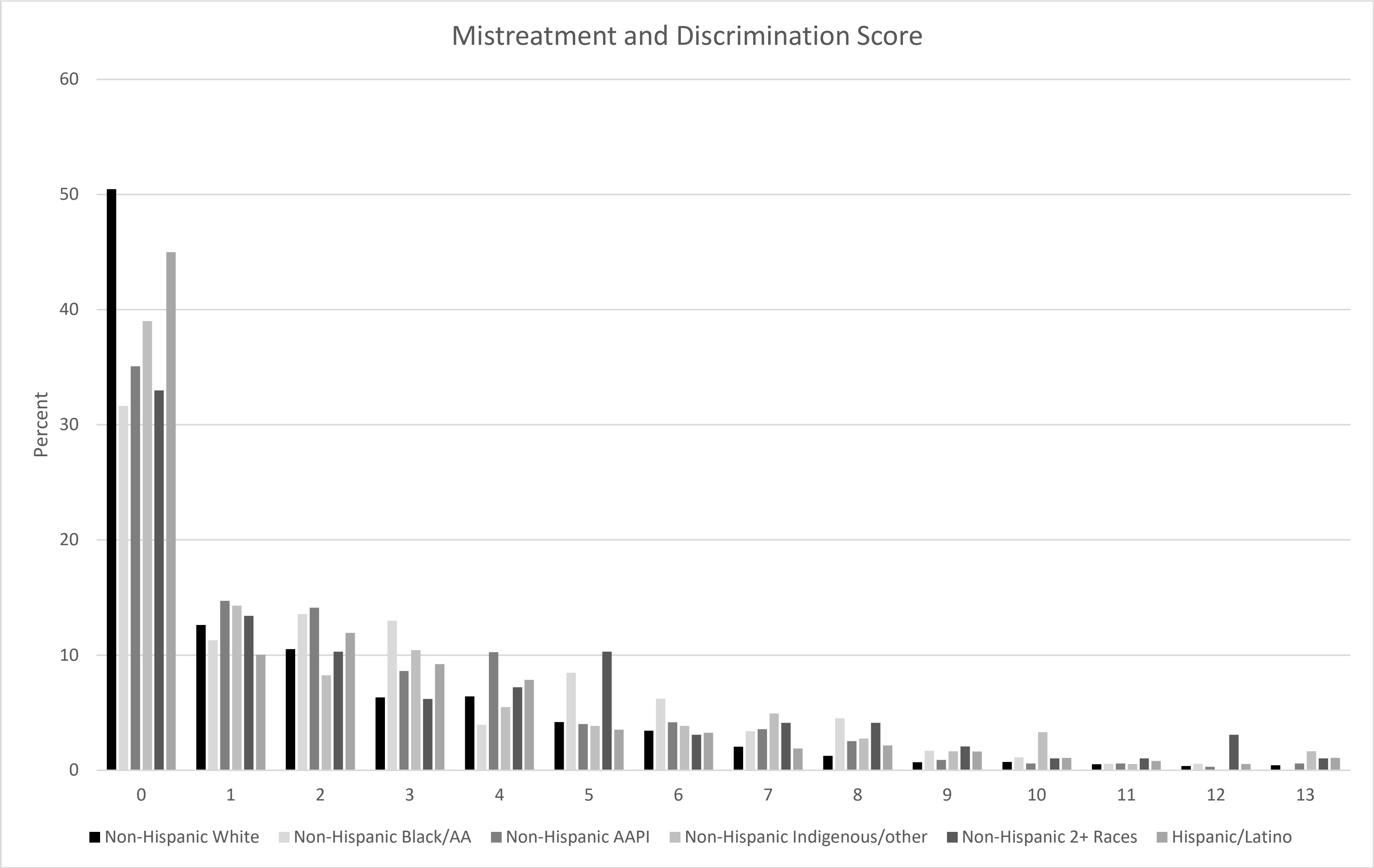

© 2022 Dyrbye LN et al. *JAMA Network Open.*

eFigure 3. Mistreatment and Discrimination Score and Mean Emotional Exhaustion and Depersonalization Scores

As mistreatment and discrimination score increased, so did the frequency of endorsing symptoms of emotional exhaustion (EE) and depersonalization (DP, both $p < 0.001$).

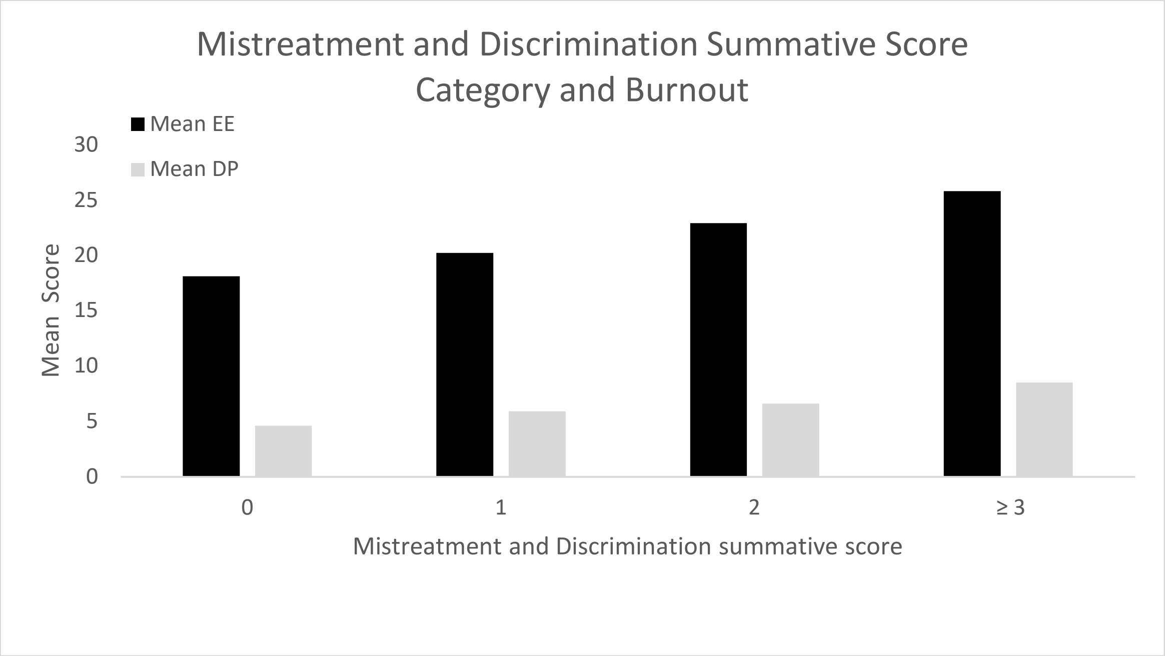

© 2022 Dyrbye LN et al. *JAMA Network Open.*

eFigure 4. Mistreatment and Discrimination Score and Burnout

As mistreatment and discrimination score increased, so did the frequency of high emotional exhaustion (EE), high depersonalization (DP) and burnout (all $p < 0.001$).

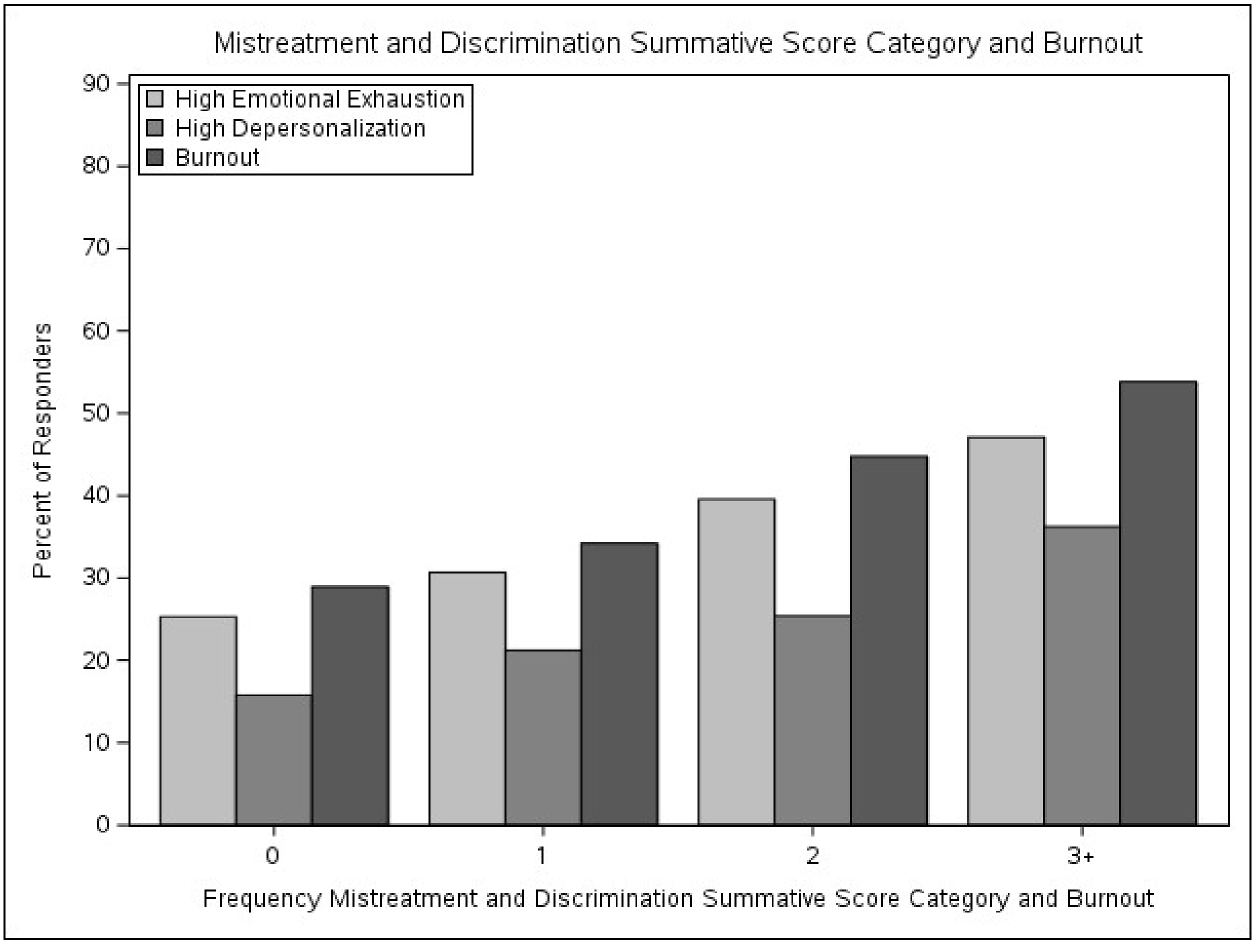

© 2022 Dyrbye LN et al. *JAMA Network Open.*
